# How Ketamine Affects Livers of Pregnant Mice and Developing Mice?

**DOI:** 10.3390/ijms18051098

**Published:** 2017-05-19

**Authors:** Hoi Man Cheung, Tony Chin Hung Chow, David Tai Wai Yew

**Affiliations:** 1 School of Chinese Medicine, The Chinese University of Hong Kong, Shatin, Hong Kong, China; chm1384@yahoo.com.hk (H.M.C.); tonychowbr@gmail.com (T.C.H.C.); 2 Hong Kong College of Technology, 2 On Shing Street, Ma On Shan, Shatin, Hong Kong, China

**Keywords:** ketamine, pregnant, liver enzyme, cell death, neonate, proliferation

## Abstract

It is well known that ketamine abuse can induce liver damage in adult addicts, but the effects of ketamine abuse in pregnant mothers on their offspring have received less attention. In this study, we investigated the effects of 5-day ketamine injections (30 mg/kg) to pregnant Institute for Cancer Research (ICR) mice during early gestation or mid-gestation on the aspartate aminotransferase (AST) and alkaline phosphatase (ALP) activities of the mothers and the offspring. We also looked into whether administering ketamine treatment to the mothers had any effects on the extent of fibrosis, cell proliferation and cell death in the livers of the newborns. No significant biochemical differences were found between treatment and control groups in the mothers. In the offspring, ketamine treatment mildly suppressed the gradual increase of hepatic AST activity in neonates during liver maturation. Measurements of hepatic ALP activity and lactic acid dehydrogenase (LDH) immunoreactivity revealed that ketamine treatment may lead to increased cell death. Proliferation of liver cells of the newborns was also retarded as shown by reduced proliferative cell nuclear antigen (PCNA) immunoreactivity in the ketamine groups. No obvious fibrosis was evident. Thus, we demonstrated that ketamine administration to pregnant mice suppressed hepatic development and also induced liver cell death of the offspring.

## 1. Introduction

In recent years, with the increase of various sorts and types of abusive drugs, researchers and authorities have been put on high alert. Scientific studies on abusive drugs have again reached an all-time high and there are now many types of abusive agents available which addicts consume; amongst these are nicotine, alcohol, marijuana, opioid, cocaine, methamphetamine and ketamine. In spite of the large number of studies on the effects of these agents on general adult addicts, their effects on fetuses and indeed even on the addicted mothers during pregnancy have received less attention. Little has been recorded except on the nervous system where cognition defects and psychiatric delays in development have been noted [1,2]. A review paper of Behnke and Smith [3] summarized the results of the limited studies on the abusive effects of nicotine, alcohol, marijuana, opiate, cocaine and methamphetamine on fetuses and newborns. There was, however, no mention of ketamine as an abusive agent. The report of Behnke and Smith suggested common, as well as diverse effects, on the prenatal and neonatal subjects subsequent to maternal substance abuse [3]. For the growth of the fetuses after maternal abuse, Behnke and Smith reported that fetal growth was retarded after abuse of nicotine, alcohol, opioid and methamphetamine, while anomalies were found only after alcohol treatment and behavioral changes were observed in the neonates of all groups [3]. For the long term effect on the children after birth, growth retardation was only apparent in the alcohol abuse group, while nicotine, alcohol, marijuana and opioid affected the behaviors of all children from mothers abusing those substances [3]. Cognitive changes and language problems also developed in the children with mothers who abused nicotine, alcohol and opioid [3]. These studies summarized by Behnke and Smith [3], on behalf of the American Academy of Pediatrics, was comprehensive, but not exhaustive, as the effects of those abusive drugs often led to new areas of inquiry as research techniques advanced. Furthermore, whether drugs like ketamine have prenatal effects or not, is yet to be explored in humans. For instance, Dong [4] has reported that ketamine used prenatally would affect the brains of the offspring of rodents, prevent neurogenesis and induce cell death. It was well documented that practically all abusive agents could, in some way, pass from mothers to their children, and in the majority of cases, via the placenta [3].

While research has been conducted on the growth of the body and the changes in the nervous system in the children with mothers on abusive drugs, there is, however, comparatively little research about the other organs of the mothers and their developing fetuses in the course of pregnancy while the mothers are on abusive drugs. It has been documented that liver damage occurred in mice models subsequent to a long-term ketamine (30 mg/kg) addiction of three to six months [5,6]. When combining ketamine treatment with a month of alcohol, the increase of glutamate oxaloacetate transaminase (GOT, also named aspartate aminotransferase, AST) in the liver was significantly more than those of ketamine alone, along with large foci of fibrosis [5]. Loss of glycogen was another principle finding of ketamine induced damage in the liver [7]. Although the toxicity in the livers of adult addicts was rather clear, in this paper we explore whether ketamine produced equivalent changes in the livers of the pregnant abusers as well as in the livers of their offspring.

## 2. Results

### 2.1. Serum Aspartate Aminotransferase (AST) Activity of Pregnant Mice

**Figure 1:**
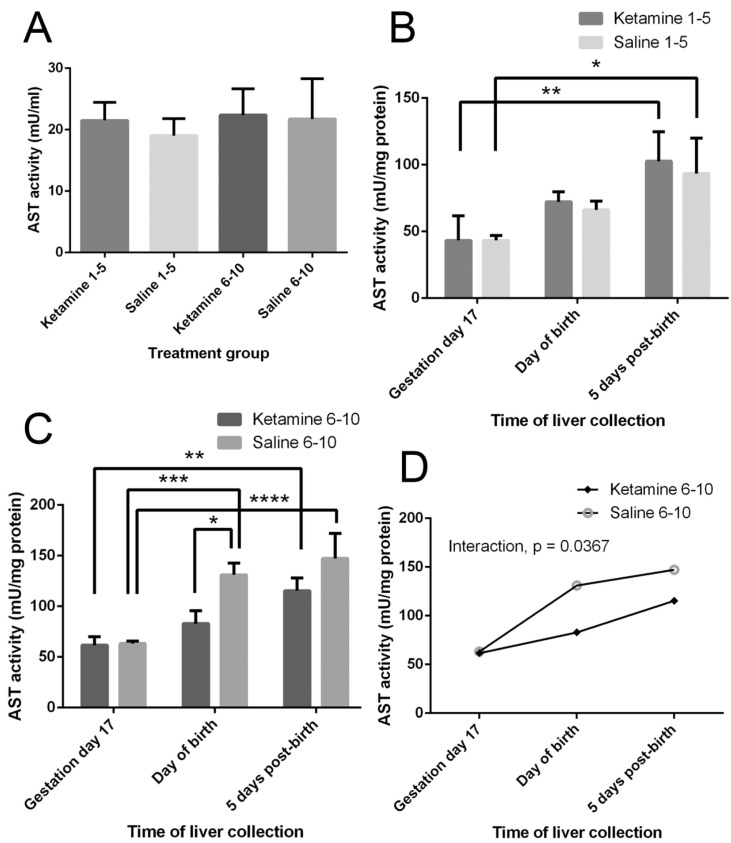
Effects of ketamine on aspartate aminotransferase (AST) activity. (**A**) Serum AST activity of pregnant mice at gestation day 17. Results were analyzed by *t*-test against respective saline control. No significant differences were observed (*p* > 0.05); (**B**) Hepatic AST activity of offspring from mothers treated on days 1–5 of gestation. Results were analyzed by two-way ANOVA followed by Tukey’s test: effect of time of liver collection, F(2,12) = 16.37 (*p* = 0.0004); effect of treatment, F(1,12) = 0.4293 (*p* = 0.5247); effect of interaction, F(2,12) = 0.12, *p* = 0.888; (**C**) Hepatic AST activity of offspring from mothers treated on days 6–10 of gestation. Results were analyzed by two-way ANOVA followed by Tukey’s test: effect of time of liver collection, F(2,12) = 38.49 (*p* < 0.0001); effect of treatment, F(1,12) = 17.54 (*p* = 0.0013); (**D**) Interaction plot of the effect of ketamine injection to the mothers at gestation days 6–10 and time of liver collection on the hepatic AST activity of offspring. Effect of interaction, F(2,12) = 4.409 (*p* = 0.0367). Values are mean ± SD, *n* = 3. * *p* < 0.05; ** *p* < 0.01; *** *p* < 0.001; **** *p* < 0.0001.

No statistically significant differences were observed in the serum AST activities of pregnant mice across different treatment groups (*p* > 0.05) (Figure 1A).

### 2.2. Liver AST Activity of Offspring

There were significant effects of the time of specimen collection (i.e., age of offspring) on the hepatic AST activities (days 1–5 groups: F(2,12) = 16.37, *p* = 0.0004; days 6–10 groups: F(2,12) = 38.49, *p* < 0.0001) (Figure 1B,C). These indicated a general increase in hepatic AST activities as the offspring grew from fetuses to 5-day neonates when the livers developed. A significant effect of treatment on the AST activity was only observed when comparing the days 6–10 groups, F(1,12) = 17.54, *p* = 0.0013 (Figure 1C). We also observed a mild but significant interaction between treatments and the time of specimen collection in the days 6–10 groups, F(2,12) = 4.409, *p* = 0.0367 (Figure 1D). In these groups, the hepatic AST activities increased from gestation day 17 to five days post-birth in the offspring from both mothers injected with ketamine and those injected with saline only. However, ketamine administration to the mothers caused a less marked increase in the hepatic AST levels in their offspring.

### 2.3. Serum Alkaline Phosphatase (ALP) Activity of Pregnant Mice

**Figure 2:**
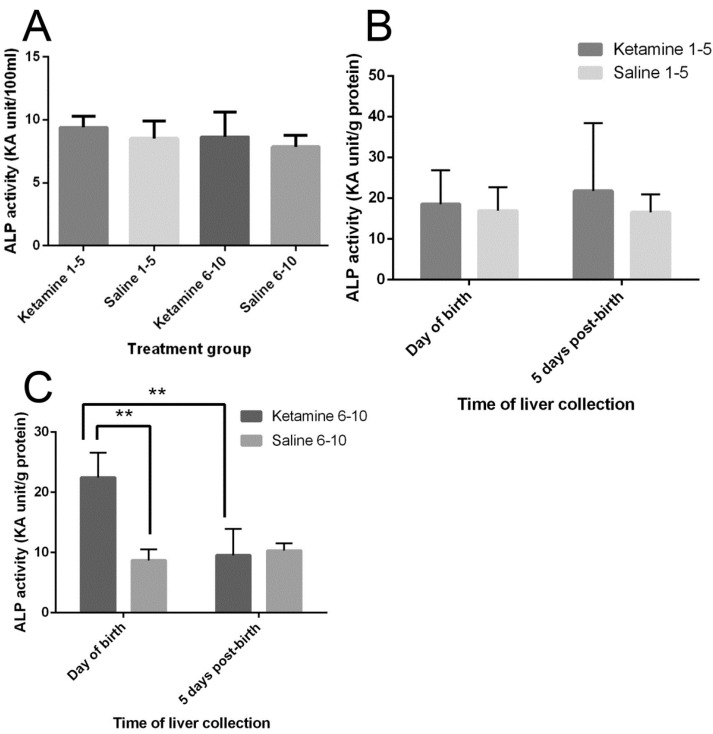
Effects of ketamine on alkaline phosphatase (ALP) activity; (**A**) Serum ALP activity of pregnant mice at gestation day 17. Results were analyzed by t-test against respective saline control. No significant differences were observed (*p* > 0.05); (**B**) Hepatic ALP activity of offspring from mothers treated on days 1–5 of gestation. Results were analyzed by two-way ANOVA followed by Tukey’s test. No significant differences were observed (*p* > 0.05); (**C**) Hepatic ALP activity of offspring from mothers treated on days 6–10 of gestation. Results were analyzed by two-way ANOVA followed by Tukey’s test. Values are mean ± SD, *n* = 3. ** *p* < 0.01.

No statistically significant differences were observed in the serum ALP activities of pregnant mice across different treatment groups (*p* > 0.05) (Figure 2A).

### 2.4. Liver ALP Activity of Offspring

In the livers of neonates of mothers injected with ketamine in the first trimester (days 1–5) of gestation, no significant difference in ALP activity was observed (*p* > 0.05) (Figure 2B). On the other hand, in the neonates from mothers treated with ketamine on days 6–10 of gestation, the hepatic ALP activity of one-day-old newborns was significantly higher than its saline control (*p* = 0.0034) as well as in the five-day-old counterparts (*p* = 0.0049) (Figure 2C).

### 2.5. Liver Histology

**Figure 3:**
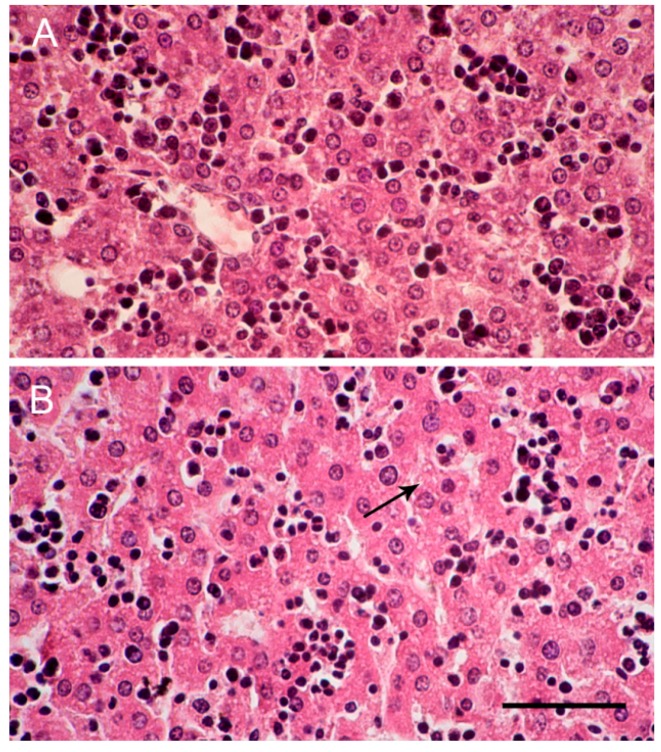
Histology of the liver cells of newborn mice (hematoxylin and eosin stain). Images are liver cells of newborn mice (**A**) at the day of birth from control mother injected with saline on days 1–5 of gestation and (**B**) five days post-birth from ketamine mother treated on days 6–10 of gestation (400× magnification). Scale bar: 50 µm. Arrow indicates fatty droplets inside cells.

Using hematoxylin and eosin histological stains, it was observed that the clusters of hepatocytes in the livers of day 1 newborn control mice were less cord-like in structure (Figure 3A). By day 5 after birth, sinusoidal space became more compact and liver cells became cord-like (Figure 3B). In both day 1 and day 5 newborn mice from ketamine-treated mothers, no remarkable degeneration was observed, though in day 5 pups from ketamine groups, there were slight fatty changes in some liver cells (Figure 3B). In both control and ketamine pups, the sinusoidal spaces were filled with Kupffer cells.

### 2.6. Sirius Red Staining of Collagen

**Figure 4:**
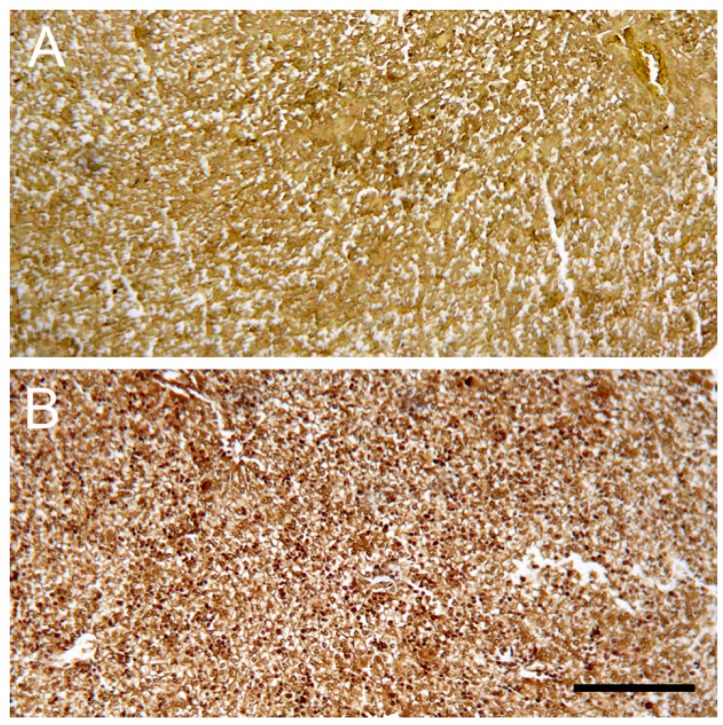
Sirius red stained liver cells of newborn mice. Images are liver cells of newborn mice at the day of birth (**A**) from ketamine mother treated on days 1–5 of gestation and (**B**) from control mother injected with saline on days 6–10 of gestation (100× magnification). Scale bar: 150 µm. No observable fibrosis was found in the livers of all groups of pups.

No obvious collagen fibers (stained red) were found in livers from all groups of newborns and five-day-old neonates (Figure 4).

### 2.7. Immunostaining of Lactic Acid Dehydrogenase (LDH)

**Figure 5:**
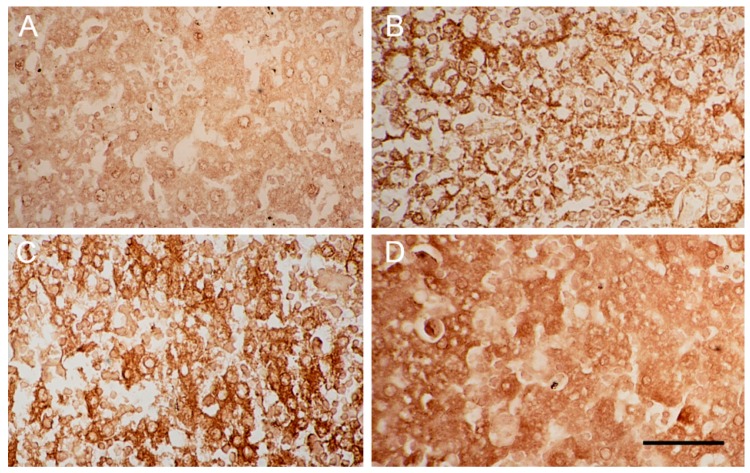
Liver cells of newborn mice with LDH immunostaining. Images are liver cells of newborn mice (**A**) five days post-birth from control mother injected with saline on days 6–10 of gestation; (**B**) at the day of birth from ketamine mother treated on days 1–5 of gestation; (**C**) at the day of birth from ketamine mother treated on days 6–10 of gestation and (**D**) five days post-birth from ketamine mother treated on days 1–5 of gestation (400× magnification). Scale bar: 50 µm.

With LDH immunohistochemistry, in the control newborn pups, there was minimal immunoreactivity (approximately 2–4% of cells were stained LDH-positive), (Figure 5A). In the pups with ketamine treated mothers, on the day of birth, 10% and 20% of cells demonstrated LDH immunoreactivity in the days 1–5 and the days 6–10 groups, respectively (Figure 5B,C). By day 5 after birth, approximately 20% of cells were LDH positive in both groups, i.e., those with ketamine mothers treated on days 1–5 of gestation and days 6–10 of gestation (Figure 5D). Data are summarized in Table 1.

### 2.8. Immunostaining of Proliferative Cell Nuclear Antigen (PCNA)

**Figure 6:**
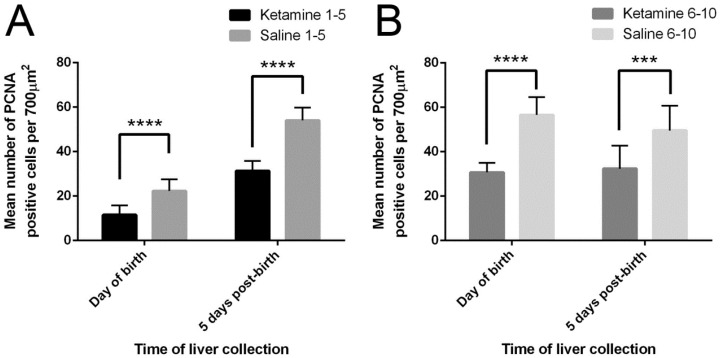
Effects of ketamine on liver cell proliferation. Average number of PCNA-positive cells in the livers of offspring from mothers treated on (**A**) days 1–5 of gestation and (**B**) days 6–10 of gestation. Results were analyzed by two-way ANOVA followed by Tukey’s test. Values are mean ± SD, *n* = 10. *** *p* < 0.001; **** *p* < 0.0001.

**Figure 7:**
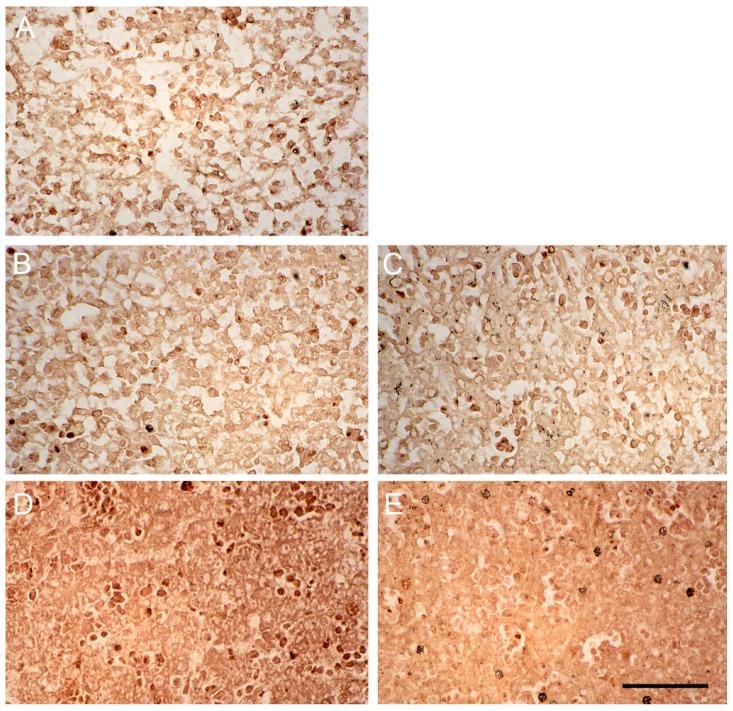
Liver cells of newborn mice with PCNA immunostaining. Images are liver cells of newborn mice (**A**) at the day of birth from control mother injected with saline on days 1–5 of gestation; (**B**) at the day of birth from ketamine mother treated on days 1–5 of gestation; (**C**) at the day of birth from ketamine mother treated on days 6–10 of gestation; (**D**) five days post-birth from control mother injected with saline on days 6–10 of gestation and (**E**) five days post-birth from ketamine mother treated on days 1–5 of gestation (400× magnification). Scale bar: 50 µm.

The numbers of liver cells from neonates stained PCNA-positive in 10 randomly selected fields (700 µm^2^ each) under the microscope (400× magnification) were counted (Figure 6). The livers of the control newborn mice from mothers injected with saline contained more PCNA-positive cells than the livers of corresponding neonates from mothers treated with ketamine (Figure 7A–E). The injection of ketamine to pregnant mothers in the 1st trimester or mid-trimester both retarded fetal liver proliferation.

## 3. Discussion

Aspartate aminotransferase (AST) is an enzyme that catalyzes the reversible interconversion between aspartate and glutamate and, as such, it is an important enzyme in amino acid metabolism. Serum AST levels may be used as a biomarker for liver injury where there is an increase in the release of the enzyme from the liver into the bloodstream. The results on the liver enzyme (serum AST) in the pregnant mice injected in the first five days of gestation versus the pregnant mice injected in mid-gestation (days 6–10) did not present any marked variation between themselves and between the control saline and experimental groups. Hence, the time of injection would not cause different deleterious effects on the livers of the mothers, provided that the durations were equal. The samples of the livers of offspring were taken out in three lots. The first lot was the fetuses at day 17 of gestation, the second lot was those of newborns, and the third lot was those of five days postnatal. When the results were tabulated, there were no differences between the control and experimental livers in AST activity in each group when the ketamine treatment to mothers was instituted from days 1–5 during gestation. However, there was an increase in the activity of AST in all groups (control and experimental) as the fetuses aged, indicating the AST amount was related to liver maturation. In the livers of the equivalent groups of fetuses, newborn and postnatal mice with mothers injected on gestation days 6–10, while AST increased along with ages as depicted above, there was an additional trend, in which AST levels in the newborn and postnatal mice demonstrated marked differences between those from mothers treated with ketamine and those from mothers injected with saline. In fact, the livers of offspring from mothers treated with saline had higher AST levels than offspring from ketamine mothers. This appears to show, that apart from the maturation of the liver, ketamine treatment in the mother might have an effect on the livers of the fetuses, newborn and postnatal mice. A significant possibility is that the injection of ketamine could have affected the maturation of the livers so that the AST values were lower in experimental specimens than in those of the control group (from mothers injected with saline). This hypothesis appears to align with former reports on fetal hepatic development of rodents and sheep [8,9,10]. If this hypothesis is accepted, then ketamine affected the liver in terms of retardation in biochemical development.

It is well known that, in addition to changes of liver enzymes, ketamine addiction for long periods of time in adult humans also contributed to other significant damage in the liver, including microscopic damages, decreased storage of glycogen and formation of sites of fibrosis in non-pregnant adults [7]. In this study, we demonstrated a decrease in proliferation ability as reflected by lower numbers of PCNA-positive nuclei per unit area of the livers in the neonates of mice with mothers treated with ketamine during pregnancy, in comparison to a control group with mothers injected with saline only. This revealed a retardation of hepatic growth in the former group. Furthermore, the retardation appeared to be evident in both newborn and five-day-old groups from mothers treated during an earlier stage (days 1–5) of gestation as well as in a later (days 6–10) stage of gestation. This retardation of growth was further compounded by the reduction of transaminase in the livers of pups with ketamine treated mothers, which all together, points to some damage in the livers of newborns with mothers on ketamine addiction during pregnancy, irrespective of whether the addiction was in the first trimester or later. On the other hand, significant differences in necrotic cell death of the liver (via LDH immunohistochemistry) were noted in the livers of both newborns and five-day-old neonates of ketamine mothers. This result pointed to cellular damage in the liver. Detectable fibrosis (via Sirius Red staining for collagen) was not found in the livers of the pups with ketamine mothers, possibly because the formation of abundant fibrotic fibers is a slow process.

From the present study, the sequence of liver damage in neonates with ketamine mothers included: (1) decrease of the transaminase AST level in the livers of the neonates with ketamine mothers, especially at day 5 postnatal; (2) enhanced cell death as demonstrated by LDH and ALP increase, bearing in mind the latter reached a new high at day 1. The logic of performing necrotic evaluation with LDH rather than apoptosis with TUNEL was that in previous studies, the effect of ketamine on the vascular system had been particularly emphasized [11]; (3) regenerative ability of the livers in the neonates with ketamine mothers went down in comparison to those from saline treated mothers, as shown by PCNA morphometry.

As the central nervous system of the pups with ketamine treated mothers is likely to be affected as well, extensive research in the future will be focused on this system which demands detailed pathological, biochemical, physiological as well as behavioral evaluations. We have to remember that some of the induced changes might not be immediately apparent in the neonatal period, but rather, in the periods of adolescence and young adulthood. This is what we are waiting to see as the neonates grow up. Furthermore, many other organs, e.g., the brain, the heart and the kidneys of the progeny have to be investigated in adulthood, specifically in terms of the pathomorphological, pathophysiological, pharmacological and biochemical changes of these areas before any conclusive statements could be made [12,13,14,15,16,17,18,19,20,21,22].

## 4. Materials and Methods

### 4.1. Animal Studies

The animal experiments were approved by the Department of Health, HKSAR government (Ref.: (17209) in DH/SHS/8/2/1 Pt.1). Eight-week old female ICR (Institute for Cancer Research, Sutton, UK) mice weighing around 25 g each were allowed to mate. On the day when the mating plug was observed, the mice were randomly assigned into four groups. For groups 1 and 2 (ketamine 1–5 and saline 1–5 groups), pregnant mice were injected with 30 mg/kg ketamine in saline or saline alone, respectively, on gestation day 1 to day 5 daily. In groups 3 and 4 (ketamine 6–10 and saline 6–10 groups), pregnant mice were injected with ketamine or saline on gestation day 6 to day 10 daily. Each group had six pregnant animals. The dosage (30 mg/kg) used in the present study was subanesthetic to ICR mice which had been previously demonstrated by our group [22]. All injections were performed subcutaneously on the back. The experimental mice were kept in the animal facility on a 12 h/12 h dark-light cycle and fed with water and standard chow ad libitum.

### 4.2. Sample Collection

In each experimental group, at least two offspring from each litter were taken at each of three different time points—day 17 of gestation, day of birth and five days postnatal. At day 17 of gestation, mothers were anesthetized by intramuscular injection of a ketamine (100 mg/kg)/ xylazine (10 mg/kg) cocktail and ~1 mL of blood was collected by cardiac puncture. Mothers were decapitated immediately afterwards. Fetuses were taken out from the uterus and their livers were collected after decapitation. Livers of newborns and five-day-old neonates were also obtained after decapitation, which were immediately snap frozen in liquid nitrogen and stored at −80 °C. Blood samples collected from mothers were allowed to clot at room temperature for 30 min and sera were obtained after centrifugation at 2000× *g* for 15 min, which were subsequently stored at −20 °C.

For histological observations, livers were dissected from newborn and five-day-old pups which were fixed in 10% neutral buffered formalin solution for at least 24 h, followed by dehydration through graded alcohols and cleared in xylene and finally embedded in paraffin.

### 4.3. Biochemical Assays

For the offspring, around 50 mg of liver specimens were weighed and homogenized in 200 µL of ice-cold phosphate buffered saline using plastic tissue grinders, followed by sonication. Clear tissue lysates were obtained from supernatant after centrifugation at 13,000× *g* at 4 °C for 10 min to remove insoluble materials. Sera from mothers and tissue lysates prepared as described above were diluted in assay buffer supplied with the AST assay kit (Abcam, Cambridge, UK) and ALP assay kit (Nanjing Jiancheng Bioengineering Institute, Nanjing, China). The assays were performed according to the manufacturers’ protocols. The calculated enzyme activities of liver samples were normalized to the protein content measured using the Pierce BCA protein assay kit (Thermo scientific, Waltham, MA, USA).

### 4.4. Histopathological Studies

Fixed liver tissues embedded in paraffin wax were cut into sections at 6 µm thickness and laid on glass slides. The sections were stained with hematoxylin and eosin for routine observation. Briefly, the tissue sections were de-waxed through xylene, graded alcohols and subsequently rehydrated in ddH_2_O. After staining in Harris hematoxylin solution for 10 min, the sections were rinsed in running tap water for 1 min before a brief immersion in acid alcohol, followed by another rinse under running tap water. Subsequently, the sections were stained with 1% eosin yellowish for 5 min, then dehydrated through graded alcohols and cleared in xylene. The stained slides were finally mounted with DPX mountant.

Tissues were stained by Sirius red staining [23] for collagen fibers. The sections were de-waxed and rehydrated as described above. The sections were then stained with Weigert’s iron hematoxylin solution for 8 min followed by a 10-min wash under running tap water. Subsequently, the sections were stained in picrosirius red solution for an hour and washed in two changes of acidified water. Finally, the stained slides were dehydrated in three changes of 100% ethanol, cleared in xylene and mounted with DPX mountant.

For immunohistochemistry, staining of proliferative cell nuclear antigen (PCNA) was used to detect the formation of new cells in the liver [24]. The presence of necrotic cells was detected by immunostaining of lactic acid dehydrogenase (LDH) [5]. To begin, tissue sections were de-waxed and rehydrated as described above. The slides were then heated to boiling point in citrate buffer using a microwave oven. After cooling down to room temperature, the slides were washed in phosphate-buffered saline with 0.05% tween 20 (PBST) for 5 min for 4 times. Sections were then incubated in methanol with 0.3% H_2_O_2_ for 10 min. After that, the slides were blocked for 2.5 h in bovine serum albumin (BSA) solution (PBS with 1% BSA and 0.1% triton X-100) and incubated in a primary antibody diluted 1:500 in BSA solution overnight at 4 °C. The antibody for PCNA was ab29 mouse monoclonal antibody [PC10] to PCNA (Abcam, Cambridge, UK) and the antibody for LDH was ab52488 rabbit monoclonal [EP1566Y] to LDH (Abcam, Cambridge, UK). On the following day, the slides were washed 4 × 5 min in PBST, incubated in secondary antibodies (Invitrogen, Carlsbad, CA, USA) diluted 1:1000 in BSA solution at room temperature for 1 h and washed again 4 × 5 min in PBST. The sections were then incubated with streptavidin–horseradish peroxidase (HRP) diluted 1:2000 (43432, Invitrogen, Carlsbad, CA, USA) in PBST for 1 h at room temperature. After washing in PBST for four times, these sections were stained in 3,3-diaminobenzidine (DAB) using a DAB peroxidase (HRP) substrate kit (Vector Laboratories, Burlingame, CA, USA). The stained slides were dehydrated through graded alcohols and cleared in xylene and finally mounted with DPX mountant.

For quantitation of immunoreactive cell densities, the numbers of positive cells were counted in a total of 10 randomly selected fields, each with a size of 700 µm^2^, from the livers of 4–6 animals from each group.

The histology of 4–6 individual liver specimens from each group of mice was analyzed. All histological figures shown in this article were representative images of the respective treatment groups.

### 4.5. Statistical Analyses

Data were analysed using Student’s *t*-test or two-way ANOVA with Tukey’s multiple comparison test (GraphPad Prism 6, La Jolla, CA, USA). Results were presented as mean ± SD. An alpha level of 0.05 or smaller was considered significantly different.

## Figures and Tables

**Table 1:** Percentage of Lactic Acid Dehydrogenase (LDH)-positive cells in the livers of neonates.

Time of Liver Collection	Ave% LDH-Positive Cells of Different Treatment Groups			
	Ketamine Mice Treated on Days 1–5 of Gestation	Control Mice Treated on Days 1–5 of Gestation	Ketamine Mice Treated on Days 6–10 of Gestation	Control Mice Treated on Days 6–10 of Gestation
Day of birth	10%	2%	20%	2%
5 days post-birth	20%	4%	20%	2%
